# Supplementary material for: Strengthening vaccination delivery system resilience in the context of protracted humanitarian crisis: a realist-informed systematic review
Source: BMC Health Serv Res. 2022 Oct 23;22:1277. doi: 10.1186/s12913-022-08653-4 (PMC9589562; doi:10.1186/s12913-022-08653-4)
Supplement: Supplementary file 1 — Additional file 1: Appendix 1. Developing a working analytical framework for the review. [file 12913_2022_8653_MOESM1_ESM.docx]

## SUPPLEMENTARY MATERIALS

### Appendix 1: developing a working analytical framework for the review

Understanding of which interventions may bolster system resilience in health, and how remains limited partly because of enduring analytical differences over the way in which resilience is conceptualised (1,2). There is, however, a degree of consensus in the health literature on the kinds of *attributes* associated with system resilience, if not necessarily the means by which these can be enhanced. They include “hard” attributes such as availability of material and human resources, and the existence of collateral pathways (i.e. the existence of multiple mechanisms through which, for example, medical products or health services can be delivered) (3,4). Effective information management is also vital although the balance between formal surveillance and softer, more immediate data from human intelligence systems in shaping system responses has emerged as an area of debate in humanitarian settings and in the context of COVID-19 responses (5,6). “Soft” attributes include features such as networking and connectivity (7). Empirical studies of resilience governance and system leadership have been few including in health, although existing health research does identify attributes including legitimacy and knowledge management as important (8–10). These attributes may derive in part from the health system and wider context in which interventions operate, but also form part of activities linked to the intervention itself.

While the ultimate goal of vaccination delivery systems can be considered to be generating improvements in vaccination coverage, and thereby population health outcomes (including mortality and morbidity from VPDs), the attributes described imply a focus on interventions that act through intermediate pathways, such as improvements in the quality of health information, integration with other services and service providers, the scale and durability of resource inputs (human, financial and others) and so on. These, and other similar outcomes, form an important focus for this review: our focus was not just on vaccination delivery endpoints (population coverage, deaths and hospitalisations averted, for example) but also on the system outputs and outcomes through which these effects were achieved.

We therefore developed a working framework to enable a layered reading of included articles, exposing:

- Context: including both the broader national context and humanitarian crisis within which the intervention implementation occurred, as well as the background state of the health system (understood with reference to the WHO health system building blocks)
- Mechanism: as a function of intervention activities on the health system building blocks, the ways in which these supported the development of recognised resilience attributes (such as collateral service pathways, strengthened networking and other features highlighted above) and ultimately the specific mechanism (whether absorptive, adaptive, or transformative) by which resilience was strengthened as a result of the intervention.
- Outcomes: vaccination-related outcomes as set out in the methods section, and as reported by included articles.

The framework outlines a loose progression from inputs to outcomes (e.g. increased vaccination coverage) but does not make explicit assertions about causal links between individual elements, about feedback loops that are, in reality, likely to apply, or about the dynamic nature of the relationship between interventions and wider context. This is because the nature of these relationships is likely to vary from intervention to intervention.

#### Appendix 1 references

1. Turenne CP, Gautier L, Degroote S, Guillard E, Chabrol F, Ridde V. Conceptual analysis of health systems resilience: A scoping review. Soc Sci Med. 2019;232:168–80.

2. Fridell M, Edwin S, Schreeb J von, Saulnier DD. Health system resilience: what are we talking about? A scoping review mapping characteristics and keywords. Int J Heal Policy Manag. 2020;9(1):6–16.

3. Barasa E, Mbau R, Gilson L. What Is Resilience and How Can It Be Nurtured? A Systematic Review of Empirical Literature on Organizational Resilience. Int J Heal Policy Manag. 2018;7(6):491–503.

4. Nuzzo JB, Meyer D, Snyder M, Ravi SJ, Lapascu A, Souleles J, et al. What makes health systems resilient against infectious disease outbreaks and natural hazards? Results from a scoping review. BMC Public Health. 2019;19(1):1–9.

5. Naimoli JF, Saxena S. Realizing their potential to become learning organizations to foster health system resilience: Opportunities and challenges for health ministries in low-and middle-income countries. Health Policy Plan. 2018;33(10):1083–95.

6. Bowsher G, Bernard R, Sullivan R. A Health Intelligence Framework for Pandemic Response: Lessons from the UK Experience of COVID-19. Heal Secur. 2020;18(6):435–43.

7. Fraccascia L, Giannoccaro I, Albino V. Resilience of Complex Systems: State of the Art and Directions for Future Research. Complexity. 2018;2018:1–44.

8. Lebel L, Anderies JM, Campbell B, Folke C, Hatfield-Dodds S, Hughes TP, et al. Governance and the capacity to manage resilience in regional social-ecological systems. Ecol Soc. 2006;11(1).

9. Blanchet K, Diaconu K, Witter S. Understanding the resilience of health systems. In: Health Policy and Systems Responses to Forced Migration. Bozorgmehr K, Roberts B, Razum O and Biddle L (eds). p. 99–117. Switzerland; Springer Verlag: 2020.

10. Blanchet K, Nam SL, Ramalingam B, Pozo-Martin F. Governance and capacity to manage resilience of health systems: Towards a new conceptual framework. Int J Heal Policy Manag. 2017;6(8):431–5.
