# Supplementary material for: Strengthening vaccination delivery system resilience in the context of protracted humanitarian crisis: a realist-informed systematic review
Source: BMC Health Serv Res. 2022 Oct 23;22:1277. doi: 10.1186/s12913-022-08653-4 (PMC9589562; doi:10.1186/s12913-022-08653-4)
Supplement: Supplementary file 2 — Additional file 2 Appendix 2. List of eligible settings and their eligibility periods. [file 12913_2022_8653_MOESM2_ESM.docx]

### Appendix 2: list of eligible settings and their eligibility periods

Using data from UN Office for the Coordination of Humanitarian Affairs (OCHA), and the humanitarian information gathering portals ReliefWeb and Humanitarianresponse.info, the following countries were categorised as eligible for inclusion as protracted crisis settings during the study period, from an initial long-list that was developed based on the World Bank country income lending group classification for 2011. The 2011 iteration was used because this fell at the mid-point of the search period, and some countries changed income classification over the period 2001-21. Note that not all listed countries were eligible for the entire study period; some were eligible only for specific time points (coinciding with periods when they were covered by a humanitarian response plan (HRP) or equivalent) – but that all countries had to have had a run of at least 5 years under a response plan of one kind or another to meet the criteria for a protracted crisis setting.

| ***Country*** | ***Eligible period(s)*** | ***Relevant response plans*** |
| --- | --- | --- |
| *Core countries (with country-specific RPs +/- relevant regional RPs of which they formed part)* | | |
| Afghanistan | Full duration of the study | Consolidated inter-agency appeals since 1995-96 (and prior to this); consecutive HRPs from 2009-2021 |
| Angola | 2001-2002 | Consolidated inter-agency appeals, 1995-2002; DRC RRP |
| Burkina Faso | 2013-2021 | Consecutive HRPs from 2013-2021 |
| Burundi | 2016-2021 | Consecutive HRPs from 2016-2021; DRC RRP |
| Cameroon | 2014-2021 | Consecutive HRPs from 2014-2021 |
| Chad | 2004-2021 | Consecutive HRPs from 2004-2021 |
| Central African Republic | 2003-2021 | Consecutive HRPs from 2003-2021 |
| Democratic Republic of Congo (DRC) | Full duration of the study | Consecutive HRPs from 1999-2021 |
| Ethiopia | 2017-2021 | Consecutive HRPs from 2017-2021 |
| Haiti | 2010-2021 | Consecutive HRPs from 2010-2021 |
| Honduras | 2014; 2021-22 | Drought emergency response 2014; HRP from 2021-22 |
| Iraq | Full duration of the study | Consolidated inter-agency appeals since at least 1995/6; consecutive Syria 3RPs 2015-2021; consecutive HRPs from 2014-2021 |
| Lebanon | 2015-2021 | Lebanon war response 2006-7; Syria 3RP 2015-2021; ERP 2021-present |
| Libya | 2011-2021 | Consecutive HRPs 2015-2021 |
| Mali | 2012-2021 | Consecutive HRPs from 2012-2021 |
| Myanmar | 2013-2021 | Consecutive HRPs from 2013-2021 |
| Niger | 2011-2021 | Consecutive HRPs from 2011-2021 |
| Nigeria | 2014-2021 | Consecutive HRPs from 2014-2021 |
| Occupied Palestinian Territories | Full duration of the study | UNRWA presence since 1949; Consecutive HRPs from 2003-2021 |
| Somalia | Full duration of the study | Consecutive HRPs from 1998-2021 |
| South Sudan | 2011-2021 (2010 if including HRP prior to independence) | Independence in 2011; Consecutive HRPs from 2010-2021 |
| Sudan | Full duration of the study | Consecutive HRPs from 1993-2021 |
| Syria | 2012-2021 | Syria HARP 2012 and subsequent domestic plans; 3RP from 2015 onwards. |
| Ukraine | 2014-2021 | Consecutive HRPs from 2014-2021 |
| Yemen | 2008-2021 | Consecutive HRPs from 2008-2021 |
| *Countries included as partners in regional refugee response plans only* | | |
| Bangladesh | 2017-2021 | Rohingya RRP 2017-21 |
| Congo | 2018-2021 | DRC RRP 2018-21 |
| Egypt | 2015-2021 | Consecutive Syria 3RPs 2015-present |
| Iran | Full duration of the study | Afghanistan RRP |
| Jordan | 2015-2021 | Consecutive Syria 3RPs 2015-present |
| Kenya | 2008-2021 | Refugee response plan 2014-2020; Emergency HRP 2008-2013; South Sudan RRP |
| Pakistan | Full duration of the study | Afghanistan RRP |
| Rwanda | 2018-2021 | DRC RRP 2018-21 |
| Tajikistan | Full duration of the study | Afghanistan RRP |
| Tanzania | 2018-2021 | DRC RRP 2018-21 |
| Turkey | 2015-2021 | Consecutive Syria 3RPs 2015-present |
| Turkmenistan | Full duration of the study | Afghanistan RRP |
| Uganda | 2018-2021 | DRC RRP 2018-21; South Sudan RRP |
| Uzbekistan | Full duration of the study | Afghanistan RRP |
| Zambia | 2018-2021 | DRC RRP 2018-21 |

Abbreviations: 3RP (Syria Crisis only) = Regional Refugee and Resilience Plan; ERP = emergency response plan; HARP = humanitarian assistance response plan; HRP = humanitarian response plan; RP = response plan; RRP = regional response plan for refugees.
