# Supplementary material for: Strengthening vaccination delivery system resilience in the context of protracted humanitarian crisis: a realist-informed systematic review
Source: BMC Health Serv Res. 2022 Oct 23;22:1277. doi: 10.1186/s12913-022-08653-4 (PMC9589562; doi:10.1186/s12913-022-08653-4)
Supplement: Supplementary file 3 — Additional file 3: Appendix 3. Sample search strategy. [file 12913_2022_8653_MOESM3_ESM.docx]

### Appendix 3: sample search strategy

Sample search structure as applied in MEDLINE:

| Ovid MEDLINE(R) ALL <1946 to September 03, 2021> | |  |
| --- | --- | --- |
|  |  |  |
| 1 | exp Immunization, Passive/ or exp Immunization Schedule/ or exp Immunization/ or exp Immunization, Secondary/ or exp Immunization Programs/ | 192796 |
| 2 | exp Vaccination/ or exp Mass Vaccination/ | 92277 |
| 3 | Vaccines/ or bacterial vaccines/ or toxoids/ or viral vaccines/ or cholera vaccines/ or diphtheria-tetanus vaccine/ or Diphtheria-Tetanus-acellular Pertussis Vaccines/ or Diphtheria-Tetanus-Pertussis Vaccine/ or Diphtheria-Tetanus Vaccine/ or Pertussis vaccine/ or Haemophilus Vaccines/ or Meningococcal Vaccines/ or BCG vaccine/ or tuberculosis vaccines/ or Heptavalent Pneumococcal Conjugate Vaccine/ or Pneumococcal vaccines/ or Papillomavirus vaccines/ or Human Papillomavirus Recombinant Vaccine Quadrivalent, Types 6, 11, 16, 18/ or Measles-Mumps-Rubella Vaccine/ or Measles Vaccine/ or Mumps Vaccine/ or Rubella Vaccine/ or Poliovirus vaccines/ or Poliovirus vaccine, inactivated/ or Poliovirus vaccine, oral/ or Viral Hepatitis Vaccines/ or hepatitis A vaccines/ or hepatitis b vaccines/ or Rotavirus vaccines/ or chickenpox vaccine/ or Influenza Vaccines/ or Typhoid-Paratyphoid Vaccines/ or Tetanus Toxoid/ or Yellow Fever Vaccine/ | 182764 |
| 4 | or/1-3 | 301617 |
| 5 | exp child/ or exp infant/ or mothers/ or women/ or pregnant women/ or female/ | 10199346 |
| 6 | 4 and 5 | 133861 |
| 7 | ((vaccinat* or revaccinat* or immunization or immunisation) adj3 (child* or infant? or newborn? or neonat* or baby or babies or toddler? or woman or women or mother?)).ti,ab. | 22878 |
| 8 | ((immunization or immunisation or vaccination) adj (rate* or coverage or uptake or adher* or complian* or drop-out or drop out or access*)).ti,ab. | 14272 |
| 9 | (((immunization or immunisation or vaccination) and (system* or service* or delivery or pathway)) adj (readiness or prepared* or responsive* or quality or safe* or resilien* or robust* or adapt* or absorb* or absorp* or transform*)).ti,ab. | 101 |
| 10 | 6 or 7 or 8 or 9 | 144976 |
| 11 | relief work/ or exp "warfare and armed conflicts"/ or refugees/ or exp disasters/ or genocide/ or ethnic cleansing/ or exp disasters/ or exp disease outbreaks/ or earthquakes/ or volcanic eruptions/ or floods/ or landslides/ or tidal waves/ or tsunamis/ or cyclonic storms/ or droughts/ or starvation/ or famine/ or disaster medicine/ | 311247 |
| 12 | (humanitarian* or protracted crisis or protracted crises or complex emergenc* or conflict-affected or conflict affected or fragile countr* or "fragile state* fragile and conflict affected" or "fragile and conflict-affected" or FCAS or insecur* or secur* or transition* countr* or internal displace* or internally displaced person* or displaced population* or mobile population* or forced migrat* or forced migrant or typhoon* or cyclone* or hurricane* or aid work* or financial crisis or economic crisis).hw,kf,ti,ab,cp. | 137200 |
| 13 | 11 or 12 | 436977 |
| 14 | Developing Countries.sh,kf. | 89235 |
| 15 | (Africa or Asia* or Caribbean or West Indies or South America or Latin America or Central America or Eastern Mediterranean or Americas or Western Pacific).hw,kf,ti,ab,cp. | 473656 |
| 16 | (Afghanistan or Bangladesh or Burkina Faso or Burundi or Central African Republic or CAR or Chad or Colombia or Democratic Republic of Congo or DRC or Eritrea or Ethiopia or Iraq or Lebanon or Mali or Myanmar or Niger or Nigeria or Pakistan or Peru or Somalia or Sudan or South Sudan or Syria or Syrian Arab Republic or Turkey or Uganda or Ukraine or Venezuela or Yemen).ti,ab. | 253763 |
| 17 | (Albania or Algeria or Angola or Argentina or Armenia or Azerbaijan or Belarus or Belize or Benin or Bhutan or Bolivia or Botswana or Brazil or Bulgaria or Cambodia or Cameroon or Chile or China or Congo or Costa Rica or Cote dIvoire or Ivory Coast or Cuba or Democratic Peoples Republic of Korea or DPRK or North Korea or Djibouti or Dominica or Dominican Republic or Ecuador or Egypt or El Salvador or Gambia or Georgia or Ghana or Guatemala or Guinea or Guinea Bissau or Haiti or Honduras or India or Indonesia or Iran or Jamaica or Jordan or Kazakhstan or Kyrgyzstan or Kyrgyz Republic or Kenya or Laos or Lao PDR or Lao or Liberia or Libya or Madagascar or Malawi or Malaysia or Maldives or Mauritania or Mexico or Mauritania or Moldova or Mongolia or Montenegro or Morocco or Mozambique or Namibia or Nepal or Nicaragua or Republic of North Macedonia or North Macedonia or Palestine or Occupied Territories or Occupied Palestinian Territories or Gaza or West Bank or Papua New Guinea or Paraguay or Philippines or Russia or Russian Federation or Rwanda or Senegal or Serbia or Sierra Leone or Sri Lanka or South Africa or Tajikistan or Tanzania or Timor Leste or Togo or Thailand or Turkmenistan or Uzbekistan or Vietnam or Western Sahara or Zambia or Zimbabwe).ti,ab. | 962412 |
| 18 | ((developing or less* developed or under developed or underdeveloped or middle income or low* income or underserved or under served or deprived or poor*) adj (countr* or nation? or population? or world)).ti,ab. | 113426 |
| 19 | ((developing or less* developed or under developed or underdeveloped or middle income or low* income) adj (economy or economies)).ti,ab. | 697 |
| 20 | (low* adj (gdp or gnp or gross domestic or gross national)).ti,ab. | 292 |
| 21 | (low adj3 middle adj3 countr*).ti,ab. | 21684 |
| 22 | (lmic or lmics or third world or lami countr*).ti,ab. | 9521 |
| 23 | 14 or 15 or 17 or 18 or 19 or 20 or 21 or 22 | 1386105 |
| 24 | 13 and 23 | 63847 |
| 25 | (10 and 24) or (10 and 16) | 6413 |
| 26 | exp health policy/ or exp health services administration/ or health services/ or child care/ or personal health services/ or community health services/ or community health centers/ or health facilities/ or ambulatory care/ or universal health care/ or child health services/ or maternal-child health services/ or telemedicine/ or preventive health services/ or "health services needs and demand"/ or workforce/ or health workforce/ or community health workers/ or exp medical informatics/ or public health informatics/ or exp population surveillance/ or behavioral risk factor surveillance system/ or epidemiological monitoring/ or exp "equipment and supplies"/ or "equipment and supplies utilization"/ or leadership/ or health education/ or healthcare financing/ or financing, government/ or financing, personal/ or induced demand/ or vaccination promotion/ or vaccination campaign/ or communication/ or exp "Health Care Quality, Access, and Evaluation"/ | 9868768 |
| 27 | (strengthening or system strengthening or health system strengthening).ti,ab. | 35858 |
| 28 | (social mobili*ation or community mobili*ation or community outreach or community engage* or community health volunteer or outreach or mobile unit or mobile medical unit or mobile team or cash transfer or cct or cash incentive or demand generat*).ti,ab. | 28371 |
| 29 | (logistic* or deliver* or supply chain* or supply-chain* or cold chain* or cold-chain*).ti,ab. | 1073065 |
| 30 | (governance or accountab* or oversight or regulat* or stewardship).ti,ab. | 2053531 |
| 31 | 26 or 27 or 28 or 29 or 30 | 12104943 |
| 32 | 25 and 31 | 4604 |
| 33 | limit 32 to (yr="2001 -Current" and (arabic or english or french)) | 3719 |
