# Supplementary material for: Strengthening vaccination delivery system resilience in the context of protracted humanitarian crisis: a realist-informed systematic review
Source: BMC Health Serv Res. 2022 Oct 23;22:1277. doi: 10.1186/s12913-022-08653-4 (PMC9589562; doi:10.1186/s12913-022-08653-4)
Supplement: Supplementary file 5 — Additional file 5: Appendix 5. Overall summary of quality appraisal results. [file 12913_2022_8653_MOESM5_ESM.docx]

### Appendix 5: overall summary of quality appraisal results

|  | | Yes | | No | | Can't tell | |
| --- | --- | --- | --- | --- | --- | --- | --- |
|  |  | n | % | n | % | n | % |
| **SCREENING QUESTIONS** | S1. Are there clear research questions? | 50 | 100% | 0 | 0% | 0 | 0% |
|  | S2. Do the collected data allow to address the research questions? | 48 | 96% | 1 | 2% | 1 | 2% |
| **1. QUALITATIVE STUDIES (n=10)** | 1.1. Is the qualitative approach appropriate to answer the research question? | 10 | 100% | 0 | 0% | 0 | 0% |
|  | 1.2. Are the qualitative data collection methods adequate to address the research question? | 8 | 80% | 0 | 0% | 2 | 20% |
|  | 1.3. Are the findings adequately derived from the data? | 9 | 90% | 0 | 0% | 1 | 10% |
|  | 1.4. Is the interpretation of results sufficiently substantiated by data? | 9 | 90% | 1 | 10% | 0 | 0% |
|  | 1.5. Is there coherence between qualitative data sources, collection, analysis and interpretation? | 9 | 90% | 0 | 0% | 1 | 10% |
| **2. RANDOMIZED CONTROLLED TRIALS (n=2)** | 2.1. Is randomization appropriately performed? | 2 | 100% | 0 | 0% | 0 | 0% |
|  | 2.2. Are the groups comparable at baseline? | 2 | 100% | 0 | 0% | 0 | 0% |
|  | 2.3. Are there complete outcome data? | 2 | 100% | 0 | 0% | 0 | 0% |
|  | 2.4. Are outcome assessors blinded to the intervention provided? | 1 | 50% | 1 | 50% | 0 | 0% |
|  | 2.5 Did the participants adhere to the assigned intervention? | 2 | 100% | 0 | 0% | 0 | 0% |
| **3. NON-RANDOMIZED STUDIES (n=5)** | 3.1. Are the participants representative of the target population? | 4 | 80% | 0 | 0% | 1 | 20% |
|  | 3.2. Are measurements appropriate regarding both the outcome and intervention (or exposure)? | 5 | 100% | 0 | 0% | 0 | 0% |
|  | 3.3. Are there complete outcome data? | 4 | 80% | 0 | 0% | 1 | 20% |
|  | 3.4. Are the confounders accounted for in the design and analysis? | 1 | 20% | 4 | 80% | 0 | 0% |
|  | 3.5. During the study period, is the intervention administered (or exposure occurred) as intended? | 4 | 80% | 0 | 0% | 1 | 20% |
| **4. QUANTITATIVE DESCRIPTIVE STUDIES (n=35)** | 4.1. Is the sampling strategy relevant to address the research question? | 34 | 97% | 0 | 0% | 1 | 3% |
|  | 4.2. Is the sample representative of the target population? | 23 | 66% | 0 | 0% | 12 | 34% |
|  | 4.3. Are the measurements appropriate? | 33 | 94% | 2 | 6% | 0 | 0% |
|  | 4.4. Is the risk of nonresponse bias low? | 13 | 37% | 5 | 14% | 17 | 49% |
|  | 4.5. Is the statistical analysis appropriate to answer the research question? | 31 | 89% | 2 | 6% | 2 | 6% |
| **5. MIXED METHODS STUDIES (n=2)** | 5.1. Is there an adequate rationale for using a mixed methods design to address the research question? | 2 | 100% | 0 | 0% | 0 | 0% |
|  | 5.2. Are the different components of the study effectively integrated to answer the research question? | 1 | 50% | 1 | 50% | 0 | 0% |
|  | 5.3. Are the outputs of the integration of qualitative and quantitative components adequately interpreted? | 1 | 50% | 1 | 50% | 0 | 0% |
|  | 5.4. Are divergences and inconsistencies between quantitative and qualitative results adequately addressed? | 1 | 50% | 0 | 0% | 1 | 50% |
|  | 5.5. Do the different components of the study adhere to the quality criteria of each tradition of the methods involved? | 1 | 50% | 0 | 0% | 0 | 0% |
